# Immunological HCV-Associated Thrombocytopenia: Short Review

**DOI:** 10.1155/2012/378653

**Published:** 2012-07-10

**Authors:** Dimitrios Dimitroulis, Serena Valsami, Paraskevas Stamopoulos, Gregory Kouraklis

**Affiliations:** ^1^Second Department of Propaedeutic Surgery, Laiko Hospital, Athens University Medical School, Sakellariou 4 Street, Alimos, 17455 Athens, Greece; ^2^Blood Transfusion Department, Areteion Hospital, Athens University Medical School, 11528 Athens, Greece

## Abstract

Infection with Hepatitis C virus (HCV) is affecting about 3% of the world's population, leading to liver damage, end-stage liver disease, and development of hepatocellular carcinoma, being thus the first indication for liver transplantation in the USA. Apart from the cirrhotic-liver-derived clinical signs and symptoms several conditions with immunological origin can also arise, such as, glomerulonephritis, pulmonary fibrosis, and thrombocytopenia. HCV-related autoimmune thrombocytopenia shows specific pathogenetic characteristics as well as symptoms and signs that differ in severity and frequency from symptoms in patients that are not HCV infected. Aim of this short paper is to estimate the epidemiological characteristics of the disease, to investigate the pathogenesis and clinical manifestation, and to propose treatment strategies according to the pertinent literature.

## 1. Introduction

Hepatitis C virus (HCV) infection is an infectious disease affecting more than 170 million individuals worldwide, representing approximately 3% of the world's population. In the United States HCV is the most common chronic blood-transmitted infection with about 1.6% of the American population being infected [1, 2]. After acute infection about 80% of these individuals develop chronic hepatitis, with many and life-threatening both hepatic and extrahepatic complications. From the hepatic complication development of liver cirrhosis, portal hypertension, end stage liver disease, and hepatocellular carcinoma are combined with high mortality and morbidity and are definitely treated with liver transplantation. The incidence of HCV-related liver dysfunction is increasing with time and HCV infection is now the leading cause of liver transplantation in the United States [3–5].

Apart from these very serious complications HCV infection is also associated with extrahepatic disorders as well as with several immune-related conditions, such as, glomerulonephritis, Sjogren syndrome, vitiligo, pulmonary fibrosis, thyroid disease, and cutaneous vasculitis [6–9]. Hematological disorders are also common in HCV-infected individuals ranging from lymphoproliferative diseases to autoimmune thrombocytopenic purpura (AITP) and autoimmune hemolytic anemia (AIHA), with the last two having probably an autoimmune underlying mechanism [6, 9, 10]. AITP is characterized by accelerated platelet destruction and variably impaired platelet production resulting in thrombocytopenia while in AIHA the red blood cells are affected. AITP is a classical paradigm of immune thrombocytopenia in infected individuals as it is associated also with HIV or Helicobacter pylori infections [11–15].

Aim of this paper is to evaluate the actual prevalence of AITP in the HCV-infected population and to distinguish probable subpopulations with different characteristics (e.g., differences between treated and untreated HCV carriers). Beside that we tried to evaluate proposed underlying mechanisms and treatment strategies as these parameters are described in the pertinent literature.

## 2. Epidemiology

The attempt to identify the exact prevalence and other epidemiological characteristics of AITP among HCV-infected individuals and especially to distinguish these characteristics between subgroups has objective difficulties, as the published studies in the pertinent literature are mainly retrospective and have several limitations. This question is also approached from two different directions. Six cross-sectional studies approached the problem by identifying serological evidence of HCV infection in patients with clinical diagnosis of AITP [16–21]. In a total of 799 patients with AITP 159 were HCV-infected (20%) with ranges from 10 to 36%. AITP patients with concomitant HCV infection were significantly older in comparison with not infected (54.9 ± 8 years versus 40.3 ± 8 years, *P* < 0.001), while there was not any distribution between sexes.

On the other hand some investigators approached the problem trying to identify AITP cases among HCV-infected individuals. Pockros et al. identified 7 AITP cases among 3440 new HCV patients over a 56-month period. The estimated prevalence of this hematologic disorder among these patients was much greater than would be expected by chance (*P* < 0.00001) [22]. Similar results provided also the study by Dufour et al., where 7 out of 4345 HCV patients fulfilled the criteria for AITP diagnosis, thus calculating the annual incidence of HCV-infection-associated AITP as being 53 cases/100000/year [23]. Nagamine et al. showed also a higher incidence of low platelet count among HCV-infected patients compared to patients infected with hepatitis B virus [10].

A very interesting and large study is that by Chiao et al., who determined the incidence of AITP and AIHA among 120691 HCV-infected and 454905 matched HCV-uninfected USA veterans who received diagnosis during the period 1997 to 2004 [24]. The results of the study showed that the overall incidence rate of AITP was higher among the HCV-infected compared with the HCV-uninfected individuals (30.2 per 100000 person years versus 18.5 per 100000 person years resp.). The one year cumulative incidence for AITP as calculated in the study among the HCV-infected population and HCV-uninfected population was 27.4 and 12.5 per 100000, respectively. In the above mentioned study there was also an attempt of differentiation in subgroups, showing that the incidence rate of AITP was slightly higher among HCV-infected individuals who received treatment in contrast to HCV-infected and untreated but not in a statistically significant manner. Interferon-*α*, a treatment regimen against HCV infection, has several serious side effects, such as, thrombocytopenia that can even lead to quit from treatment [25]. These treatment adverse effects could explain these results.

## 3. Pathogenesis Symptomatology 

Several pathogenic mechanisms have been proposed for AITP related to chronic HCV infection. One potent mechanism is the specific bind between HCV and human CD81 receptor on the platelet membrane, thus causing auto-antibody production against this complex leading to phagocytosis of platelets [26]. Beside that HCV infection causes a dysregulation of the host immune system stimulating nonspecific autoimmunity or triggering the production of specific autoantibodies against platelet membrane glycoproteins, there has been a controversy in the pertinent literature regarding the published data with respect to the presence of these specific antibodies in patients with HCV-related AITP [10, 22, 27]. Though, in one report 66% of patients infected with HCV had detectable antiplatelet glycoprotein antibodies. However, the presence of these antibodies did not have any clinical significance and did not affect the platelet count [28].

There a further mechanism has also been proposed by which HCV can directly affect megacaryocytes and therefore causes their depletion. The fact that both AIHA and AITP are autoimmune hematological disorders but HCV infection mainly causes AITP to support the hypothesis that beside a generalized autoimmune reaction there must be a specific antiplatelet autoimmune reaction. This hypothesis has led Chiao et al. to the proposal that AITP in the setting of HCV infection should be renamed as “HCV-related thrombocytopenia” in accordance to “HIV-related thrombocytopenia” [24]. At this point it should be mentioned that HCV-infected patients have further reasons to develop thrombocytopenia, such as, liver damage, impaired thrombopoietin production, portal hypertension, splenomegaly, or treatment adverse effects.

Signs and symptoms of AITP in the setting of HCV infection are more or less similar in comparison with noninfected individuals, but they are presented with differentiations in severity and frequency. So Rajan et al. published a study where symptoms and signs of thrombocytopenia were less frequent in HCV-positive patients with AITP than in HCV not infected but major bleeding was more frequent in infected individuals (25 versus 10%, *P* = 0.0059) [29]. This finding was also verified in the study of Chiao et al., where 60% of the HCV-infected patients had at least one episode of bleeding, epistaxis, or purpura [24]. On the other hand Pawlotsky et al. could not demonstrate any differences between these two groups. This study, however, had limitations, such as, evaluating patients with very low platelets count [20]. 

## 4. Treatment

Before choosing the optimal treatment modality for HCV-related AITP one should take into consideration two major parameters, raising thus a difficult therapeutic challenge. First these patients have a double disorder, and the attempt to cure one of them could enhance progress of the other. In addition timing to start treatment should include the fact that these patients have less frequent symptoms, but they bleed more severe. Beside these two parameters HCV-associated thrombocytopenia might be related to immune and/or toxic mechanisms as well as impaired platelet production as described in the section of pathogenesis. So it is often difficult to estimate the correct diagnosis for the low platelet count in order to provide optimal treatment strategies. Although there are no specific guidelines regarding treatment of AITP in HCV-infected patients intravenous immunoglobulin (IVIg) is first-line treatment regimen, similar to noninfected patients [30]. Here we should point out that unlike in primary ITP the use of steroid therapy in HCV-related thrombocytopenia was never popular due to worsening of liver function tests, rising viral load, and in some cases even causing an elevation in serum bilirubin [31]. Dufour et al. demonstrated in their study that response rate to steroids was significantly lower in HCV-positive than HCV-negative patients (*P* = 0.02). Moreover, it is recommended in the same study that steroid treatment should be avoided as much as possible as this can increase viral load and develop liver damage [23]. This later study shows similar results with previous analyses. Furthermore, in one Japanese study none of the 10 HCV-positive patients treated with prednisone achieved any response [32]. Rajan et al. found that treatment with IVIg was proven effective in increasing platelet counts in 90% of cases of HCV-infected patients but could not achieve long-term response for these patients [29]. Furthermore, patients who are Rh positive, who do not present with severe anemia and still preserve their spleens are also candidates for anti-RhD treatment. The duration of response to anti-RhD therapy is usually longer in comparison to IVIg treatment but can occasionally result in mild hemolytic anemia [33, 34].

After failure or long-time dependence to steroids or the above-mentioned classical forms of conservative treatment splenectomy is proposed as second-line therapy in several studies. Response to splenectomy is similar for both the HCV-infected and for the HCV seronegative patients [16, 32]. At this point it should be mentioned that, before proposing splenectomy as second-line therapy, we should very carefully examine the status of the patient's liver function, the stage of probable liver cirrhosis and the presence of portal hypertension or portal vein thrombosis.

Apart from classical forms of first- and second-line therapeutic proposals for HCV-related AITP further regimens have been used for selected cases. Pockros et al. reported remarkable responses under cyclophosphamide in two severe AITP cases. Due to its toxicities, though, cyclophosphamide should be limited to severe and refractory cases [22]. Dufour et al reported in their study that two out of four patients with relapsing AITP despite steroids and IVIg experienced a sustained response after rituximab treatment [23]. Rituximab is a monoclonal antibody reacting with the CD20 antigen which is present on B cells. Efficacy of this antibody has been reported in several autoimmune conditions, such as refractory severe AITP associated with HCV infection or HCV-related-mixed cryoglobulinemia [35–37]. However, due to its immunosuppressive properties rituximab should be proposed only for refractory or associated with severe bleeding cases. Further immunosuppressive agents that have also been used are azathioprine, cyclosporine A, and mycophenolate mofetil [38].

Antiviral therapy was also proposed in several studies for nonresponders to classical regimens. Approximately half of adult patients with HCV-related AITP treated with interferon-*α* responded with a rise in platelet count. Furthermore, responders to IFN-*α* could be distinguished from nonresponders by a decrease of the HCV RNA load and hepatic enzymes. Iga et al. reported significant improvement of platelets counts in twelve HCV-infected patients who were complete responders to IFN-*α* treatment, but no raise in the platelet count was seen in patients where IFN-*α* therapy failed as estimated by the viral load [27, 39].

New prospective in the treatment of HCV-related AITP is given with the use of thrombopoiesis-stimulating agents. Eltrombopag and romiplostim are small molecule thrombopoietin (TPO) receptor agonists proven to be effective in raising platelet count in patients with relapsing or refractory ITP [40, 41]. The response rate of the patients treated with high dose of eltrombopag was 70–80% compared with 11% response rate that was seen in patients who received placebo. Furthermore, McHutchison et al. described a rise in the number of platelets in HCV-infected patients with AITP to that point that these patients were eligible to continue antiviral chemotherapy without any dose reduction or interruption [42]. A third confirmatory study was performed by Bussel et al. comparing for a period of six weeks 50 mg of eltrombopag against placebo. Beside the encouraging response rates of this study some patients developed significant liver enzyme elevations requiring cessation of medication. Hepatotoxicity has been reported in patients receiving eltrombopag. In these cases discontinuation of the drug is advised. It is worth mentioning that to date hepatotoxicity has not been observed in patients treated with romiplostim [34]. A further concern regarding adverse effects of these new agents is the development of venous thromboembolic events (VTEs). Nevertheless no significant increase in incidence of VTEs has been reported in patients who received eltrombopag or romiplostim compared with the incidence of this phenomenon in ITP patients. These events also were not related to the number of platelets. However, these agents should be used with caution in patients with predisposing factors for arterial or venous thromboembolism [43]. Another worrisome safety issue of TPO mimetics is the development of bone marrow fibrosis with increased reticulin deposition. Reticulin fibrosis appears to be a rare, dose depended, and reversible side effect of TPO mimetics that resolves after discontinuation [34].

As a conclusion one could say that the multifactorial pathogenesis of HCV-related autoimmune thrombocytopenia is much more reflected on the option for the best treatment modality. A clear diagnosis is sometimes difficult to be made and in these cases the treatment regimens are controversial. Further studies are needed as new pharmacological agents in combination with former conservative and interventional therapeutic strategies should fulfill a double purpose: raise platelet count and allow continuation of antiviral treatment.
